# Remote, Smart Device-Based Cardiac Rehabilitation After Myocardial Infarction: A Pilot, Randomized Cross-Over SmartRehab Study

**DOI:** 10.1016/j.mcpdig.2024.06.001

**Published:** 2024-06-20

**Authors:** Peter Wohlfahrt, Dominik Jenča, Vojtěch Melenovský, Jolana Mrázková, Marek Šramko, Martin Kotrč, Michael Želízko, Věra Adámková, Francisco Lopez-Jimenez, Jan Piťha, Josef Kautzner

**Affiliations:** a Department of Preventive Cardiology, Institute for Clinical and Experimental Medicine, Prague, Czech Republic; b Department of Cardiology, Institute for Clinical and Experimental Medicine, Prague, Czech Republic; c Experimental Medicine Centre, Institute for Clinical and Experimental Medicine, Prague, Czech Republic; d First Medical School, Charles University, Prague, Czech Republic; e Third Medical School, Charles University, Prague, Czech Republic; f Department of Cardiovascular Medicine, Mayo Clinic College of Medicine, Rochester, MN; g Medical and Dentistry School, Palacký University, Olomouc, Czech Republic

## Abstract

**Objective:**

To evaluate the effect of smart device-based telerehabilitation on Vo_2peak_ in patients after myocardial infarction.

**Patients and Methods:**

This was a pilot, single-center, randomized, cross-over study with a 3-month intervention. One month after myocardial infarction, patients had cardiopulmonary exercise testing and a 6-minute walking test (6MWT) and were randomly assigned 1:1. In the intervention group, patients received a smartwatch to track the recommended number of steps, which was individualized and derived from the 6MWT. A study nurse telemonitored adherence to the recommended number of steps a day. In the control group, 150 minutes a week of moderate-intensity physical activity was recommended. After 3 months study arms were crossed over, and study procedures were repeated after 3 months.

**Results:**

Between June 1, 2019, and February 28, 2023, 64 patients were randomized, of which 61 (aged 51±10 years, 10% women) completed the study. Overall, the smart device-based telerehabilitation led to 2.31 mL/kg/min (95% CI, 1.25-3.37; *P*<.001) Vo_2peak_ increase compared with the control treatment. Furthermore, there was a significant effect on weight (−1.50 kg; 95% CI, −0.39 to −2.70), whereas the effect on the 6MWT distance (4.7 m; 95% CI, −11.8 to 21.1) or Kansas City Quality of Life questionnaire score (0.98; 95% CI, −1.38 to 3.35) was not significant.

**Conclusion:**

Smart device-based cardiac rehabilitation may be a promising alternative for patients unable or unwilling to attend in-person cardiac rehabilitation.

**Trial Registration:**

clinicaltrials.gov Identifier: NCT03926312

After myocardial infarction (MI), exercise training is a cornerstone of cardiac rehabilitation (CR), which reduces cardiovascular disease (CVD)-related mortality and hospital readmissions and improves quality of life.^1^^,^^2^ Independently of baseline functional status, improvement in cardiorespiratory fitness is associated with lower mortality risk in patients with and without cardiac disease.^3^ Despite proven benefits and class I recommendation in the guidelines,^4^^,^^5^ only one-quarter of eligible patients attend even 1 session of CR in the United States and 5% worldwide.^6^^,^^7^ There are several barriers that preclude delivery of CR after MI. These include insufficient access to CR centers, noncompliance of referring physicians and patients, CR staffing shortages and low socioeconomic status, and cost. Although The Million Hearts Cardiac Rehabilitation Collaborative has set a target to increase cardiac rehabilitation utilization to 70% of all eligible individuals,^8^ current CR programs in the United States can only accommodate approximately 47% of all eligible patients.^9^ On the global scale, access to CR is even worse. According to the Global Audit of Cardiac Rehabilitation, CR is available in only 111 of the 203 (54.7%) countries globally, with many countries with high burden having no (eg, in Africa) or minimal CR (eg, India, China, and Czech Republic) programs.^10^ Thus, novel models of CR delivery are needed.

Telemedicine programs are emerging as promising alternatives to current in-person CR programs. Utilization of wearable electronics in patients after MI may expand access to CR and eliminate disparities in CR services. Furthermore, remote intervention is not limited to CR working hours, which provides more flexibility and decreases time stress to used patients. However, current evidence on the effect of home-based cardiac telerehabilitation (CTR) on functional capacity is mixed. As recently recognized by the Science Advisory from the American Heart Association, several gaps need to be addressed before telerehabilitation can be implemented into everyday clinical practice.^11^

One of the important questions to be answered is physical activity prescription in telerehabilitation programs. Walking is the most accessible way of exercise that requires no special equipment, and most people can get started immediately. Furthermore, a daily step number has a marked impact on mortality and CVD-related morbidity.^12^

The present pilot, single-center, cross-over randomized study aimed to evaluate the effect of increasing the number of steps a day using a wearable device connected to a smartphone over a 3-month period on functional capacity status measured by Vo_2peak_ among patients after MI. The secondary outcomes included the effect on weight, 6-minute walking distance, glycated hemoglobin (HbA1c) level, and quality of life measured by the Kansas City Quality of Life questionnaire (KCCQ).

## Patients and Methods

This was a single-center, randomized, cross-over, pilot superiority clinical trial between June 1, 2019, and February 28, 2023, in which 64 patients were randomized, of which 61 (aged 51±10 years, 10% women) completed the study. The trial protocol was approved by the local ethical committee. Informed written consent was obtained from all participants. This study followed the Consolidated Standards of Reporting Trials reporting guidelines. The study was registered with clinicaltrials.gov (NCT03926312).

### Inclusion and Exclusion Criteria

Patients aged 18 years and older were recruited by the study nurse at the time of hospitalization for MI. Physical inactivity before MI defined as the absence of moderate to vigorous exercise for at least 30 minutes for 5 or more days a week and declined participation in center-based CR were required as inclusion criteria. Patients with advanced heart failure (The New York Heart Association classification - NYHA IIIB-IV), with planned coronary artery revascularization or other major operation during the next 12 months, or with the inability to walk for any reason, patients with a life expectancy less than 12 months, and pregnant women were considered ineligible for this study.

### Study Procedures

At the time of hospital discharge or within a few days after discharge, all patients received a smartwatch capable of tracking the daily number of steps and a smartphone to send recorded data from the smartwatch into the hospital information system. During the first month, no specific advice on physical activity was given. This period was used to test the patient’s ability to operate the devices. One month after MI, patients were invited for the first study visit (V1), during which a 6-minute walking test (6MWT), symptom-limited treadmill cardiopulmonary exercise testing (CPET), KCCQ questionnaire, and blood test for HbA1c level were performed. Afterward, patients were randomized in fixed blocks of 4 with a 1:1 ratio to the interventional arm or control arm using a computerized system. In the intervention arm (CTR), 2 patient-specific walking targets were set for each patient: (1) a total number of steps a day and (2) a number of steps at a fast walking pace similar to the 6MWT pace. An individualized total number of steps was derived from the 6MWT. The total steps number was calculated by the formula 0.9 × 5 × 6MWT number of steps, of which 25% were advised to be during brisk walk. We created this formula based on an expectation of at least 30 minutes of active minutes a day. Thus, the 6-minute number of steps was multiplied by 5. Because the 6MWT is performed at a maximal walk pace, we applied a 90% correction factor to this formula.

Study nurse monitored adherence with the training plan using data automatically uploaded into the hospital information system. Low adherence was defined as the number of steps below 80% of the recommended on 3 successive days. In the case of low adherence, the study nurse gave a motivational call to the patient.

In the control group, patients did not have the study watch and cell phone, and the guidelines recommended at least 150 minutes a week of moderate-intensity exercise was advised.^13^ After 3 months, second study visit (V2) was done, during which 6MWT, CPET, KCCQ, and blood tests were repeated. To compare the effect of delayed intervention with that of early intervention, study groups were crossed over at the second visit. Patients in the initial intervention group returned the study watch and cell phones and were instructed to comply with the 150 minutes a week of moderate-intensity exercise recommendation. The third study visit (V3) was done 3 months after the second visit, with the same examinations performed as during previous study visits.

### Study End Points

The primary end point of the study was the difference between the CTR and the control group in Vo_2peak_ change after 3 months of the intervention. The CPET reading was done by a single researcher (D.J.) blinded to group assignment. The secondary end points included the change in 6MWT, body weight, HbA1c level, and KCCQ score.

### Statistical Analyses

Descriptive statistics are reported as means ± SDs, medians (interquartile range), or frequencies (percentages). On the basis of the 2.5-mL/kg/min definition of clinically significant Vo_2peak_ increase used in a previous study,^14^ we projected an approximately 10% Vo_2_ increase with an expected 2.5±3.5 mL/min/kg Vo_2peak_ increase in the CTR group and a 0±3.5 mL/min/kg Vo_2peak_ change in the control group after 3 months. At 80% power and α of .05, 29 patients in each group were needed. Considering a 10% dropout, this study required enrollment of 32 patients in each group.

A generalized linear mixed model for repeated measures was used with intervention (CTR vs control) and time analyzed as fixed effects, with identity link and random subject effect, and adjusted for baseline value. Because the individual patient is analyzed as a random effect, this model also accounts for positive correlations of repeated measurements from the same patient. Estimated means with 95% CI are presented. The overall intervention effect was calculated as the difference between the intervention (V2 vs V1 for the intervention-first group and V3 vs V2 for the control-first group) and control periods (V3 vs V2 for the intervention-first group and V2 vs. V1 for control-first group).

All statistical tests and CIs were 2 sided with a significance level of .05. Statistical analyses were conducted with SPSS version 25.0 (IBM).

## Results

**Figure 1:**
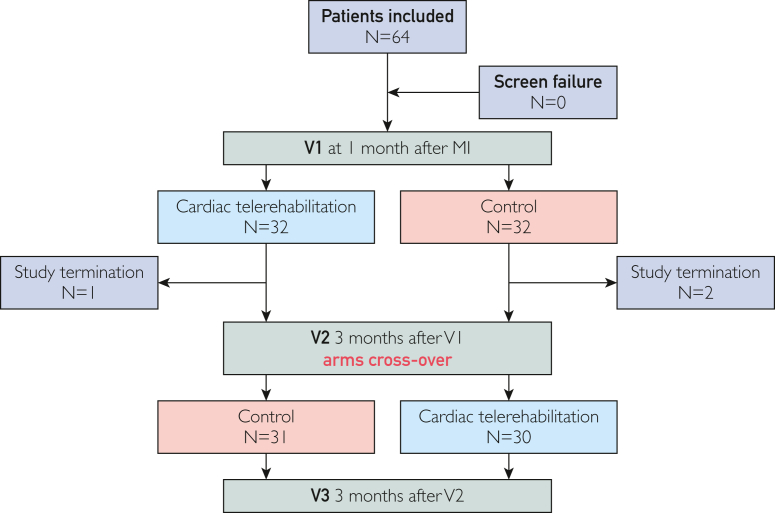
Consort diagram. MI, myocardial infarction; V1, first study visit; V2, second study visit; V3, third study visit.

From June 19, 2019, to February 13, 2023, we included 64 patients in the study. A Consolidated Standards of Reporting Trials flowchart of the trial is presented in Figure 1. During the trial, 2 patients initially randomized to the control group and 1 patient randomized to the intervention group prematurely terminated the study at their request. Reasons for study termination included newly diagnosed severe diabetic neuropathy in 1 patient and worsening of arthrosis in 2 patients. The demographic characteristics of 61 patients completing the study by initial randomization are summarized in Table.

**Table:** Population Demographic Characteristics by Study Groups

	Intervention-first (N=31)	Control-first (N=30)
Age (y)	51.6±10.2	51.2±10.5
Male sex, n (%)	28 (90%)	27 (90%)
Weight (kg)	96.8±17.4	97.0±16.7
BMI (kg/m^2^)	29.9±4.2	30.5±4.3
Diabetes history	2 (7%)	3 (10%)
Arterial hypertension history	14 (45%)	12 (40%)
STEMI, n (%)	20 (65%)	22 (73%)
Killip I, n (%)	25 (81%)	26 (87%)
Direct PCI, n (%)	30 (97%)	29 (97%)
EF at discharge (%)	46.6±8.7	49.3±10.5
EF≤40% at discharge, n (%)	10 (32%)	7 (23%)
Smoking		
Nonsmoker, n (%)	12 (39%)	10 (33%)
Current smoker, n (%)	13 (42%)	16 (53%)
Exsmoker, n (%)	6 (19%)	4 (13%)
Discharge medication		
β-blockers, n (%)	26 (84%)	25 (83%)
ACEi/ARB, n (%)	24 (77%)	22 (73%)
Statin, n (%)	31 (100%)	30 (100%)
Baseline Vo_2peak_ (mL/kg/min)	22.6±5.6	23.2±5.1
Baseline Vo_2peak_≤80% predicted	14 (45%)	12 (40%)
HbA1c (mmol/mol)	41.0±6.0	43.6±10.4

Abbreviations: ACEi/ARB, angiotensin-converting enzyme inhibitor/angiotensin receptor blocker; BMI, body mass index; EF, ejection fraction; HbA1c, glycated hemoglobin; PCI, percutaneous coronary intervention; STEM1, ST-segment elevation myocardial infarction.

### Primary Outcome

**Figure 2:**
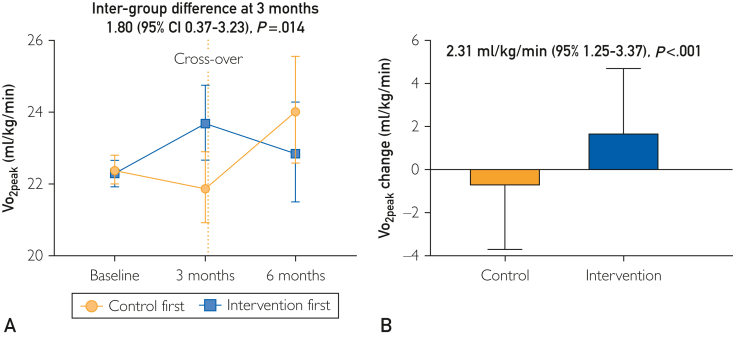
Changes in Vo_2peak_ over time (A) and by study groups (B).

After 3 months of the intervention, the Vo_2peak_ increased in the intervention group, with an intergroup difference of 1.80 mL/kg/min (95% CI, 0.37-3.23; *P*=.014) (Figure 2A). There was no statistically significant difference (*P*=0.29) in Vo_2peak_ increase by the intervention when starting early (first 3 months) or later (second 3 months).

In an analysis grouped over the whole study period, the Vo_2peak_ increased by 2.31 mL/kg/min (95% CI, 1.25-3.37; *P*<.001) (Figure 2B). There was a significant interaction by baseline functional capacity (*P*=.03). Patients with baseline Vo_2peak_ less than 80% of predicted value had larger Vo_2peak_ increase (2.92 mL/kg/min; 95% CI, 1.57-4.27) when compared with patients with normal functional capacity (Vo_2peak_ increase; 1.86 mL/kg/min; 95% CI, 0.31-3.40). There was no interaction by sex (*P*=.49) or age older than 60 years (*P*=.99). Although, numerically, the increase in Vo_2peak_ was larger in individuals with ejection fraction (EF) less than or equal to 40 (increase by 3.52 mL/kg/min; 95% CI, 1.37-5.68) when compared with individuals with EF greater than 40% (increase by 1.84 mL/kg/min; 95% CI, 0.61-3.07), the interaction in the treatment effect by EF groups was not significant (*P*_interaction_=.16) (Supplemental Figure, available online at https://www.mcpdigitalhealth.org/).

### Secondary Outcomes

**Figure 3:**
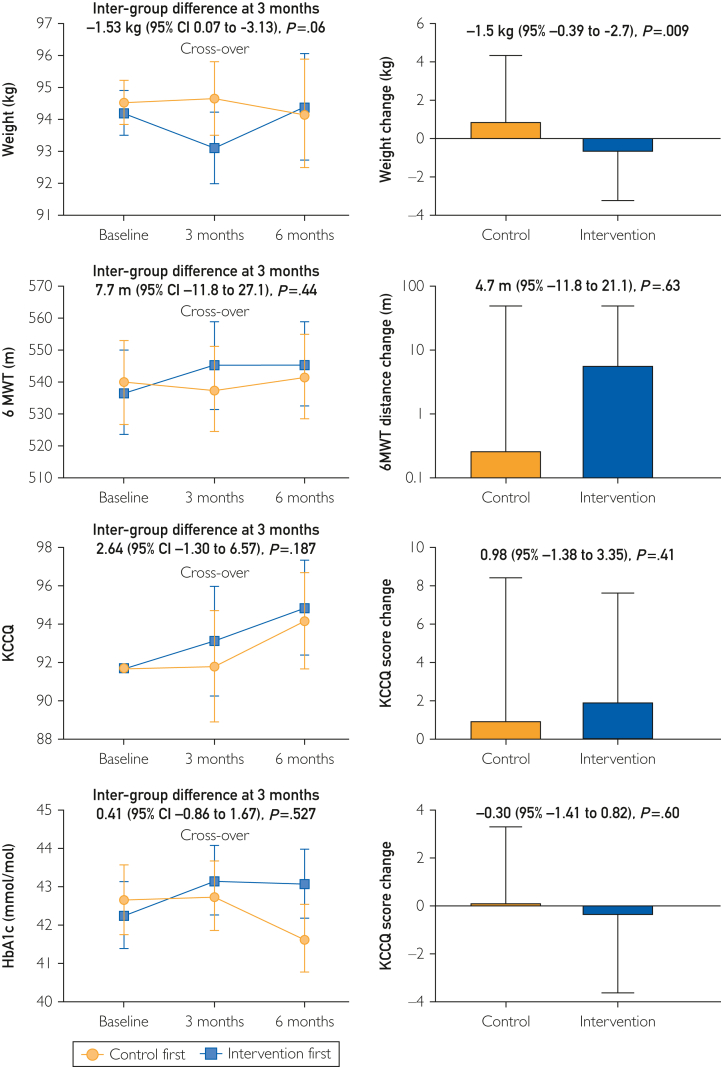
Changes in secondary outcomes. HbA1c, glycated hemoglobin; 6MWT, 6-minute walk test; KCCQ, Kansas City Quality of Life questionnaire.

The intervention did not affect submaximal exercise capacity evaluated by the 6MWT, with similar 6MWT distance at 3 months (intergroup difference, 7.7 m; 95% CI, −11.8 to 27.1; *P*=.44) and neutral overall effect (difference between control and intervention group, 4.7 m; 95% CI, −11.8 to 21.1; *P*=.63) (Figure 3).

There was a positive effect of the intervention on weight, with a borderline difference at 3 months (−1.53 kg; 95% CI, 0.07 to −3.13; *P*=.06), but a significant overall effect (−1.50 kg; 95% CI, −0.39 to −2.70; *P*<.01). Heart failure-related quality of life evaluated by the KCCQ was not affected by the intervention, with no significant difference at 3 months (2.64; 95% CI, −1.30 to 6.57; *P*=.19), and no overall difference (0.98; 95% CI, −1.38 to 3.35; *P*=.41). Glycated hemoglobin level was not affected by the intervention with similar values at 3 months (intergroup difference, 0.41; 95% CI, −0.86 to 1.67; *P*=.53) and similar overall changes (between-group difference, −0.30; 95% CI, −1.41 to 0.82).

## Discussion

This pilot study found that among patients after MI not attending traditional CR, a 3-month CTR program based on increasing the number of steps a day is beneficial in increasing functional capacity and weight reduction. The effect of the intervention was larger in patients with decreased functional capacity and Vo_2peak_ at baseline below 80% of the predicted value. Thus, these pilot data suggest that CTR based on walking may be a valuable alternative to patients refusing traditional rehabilitation programs.

The Vo_2peak_ increase by 2.31 mL/kg/min (95% CI, 1.25-3.37) observed in our study is similar to that described after in-person CR (2.52; 95% CI, 2.32-2.72).^15^ Furthermore, the 10% Vo_2peak_ improvement is beyond the 5.9% coefficient of variation for Vo_2peak_ assessment^16^ and, thus, can be classified as genuine. From the clinical point of view, the 2.3 ml/kg/min is highly clinically relevant. In a previous study, each 1-mL/kg/min increase in Vo_2peak_ was associated with an 11%, 15%, and 16% reduction in all-cause, CVD-related, and cancer-related mortality, respecitvely.^17^ Thus, the anticipated 25%-30% decrease in mortality associated with our intervention is in agreement with the reported 26% reduction in CVD-related mortality associated with in-person CR.^2^ To put the effect magnitude into perspective, similar mortality benefit has percutaneous coronary intervention in patients with MI.^18^

Although the intervention was individualized with the recommended number of steps based on the 6MWT number of steps, the effect of our intervention differed by baseline functional status. Patients with normal functional status defined by baseline Vo_2peak_ greater than 80% of predicted value had a lower increase in Vo_2peak_ than patients with decreased functional status at baseline. A possible explanation for this observation is an insufficient level of exercise intensity by walking. Previous studies have shown that vigorous-intensity exercise is more effective at increasing Vo_2peak_ than moderate-intensity exercise, even when accounting for exercise duration.^19^ This is true for healthy individuals, in whom high-intensity interval training has a larger effect on Vo_2peak_ than endurance training.^20^ However, in patients with cardiac dysfunction, the effect of high-intensity interval training on Vo_2peak_ was similar to the moderate continuous training.^14^^,^^21^ Thus, our intervention based on walking is particularly useful in patients with decreased functional capacity after MI. A higher intensity exercise may be needed in patients with preserved functional status after MI.

Previous studies have described lower benefits of CR if the beginning is delayed, when compared with early intervention^22^^,^^23^ However, in this study, the effect on Vo_2peak_ was similar in patients starting with the intervention 1 and 4 months after MI. This discrepancy may be explained by a selection bias in observational studies, with different motivation and comorbidity status in patients enrolled at different time points to CR.

A surprising observation may be the absence of 6MWT improvement, despite Vo_2peak_ improvement. This may be explained by an insufficient power of the study or by insufficient effectiveness of the intervention to improve submaximal functional capacity.

We have to acknowledge several limitations. Because this was a pilot trial, the study was underpowered for secondary outcomes. Thus, a larger adequately powered study needs to answer these questions. Because this was a single-center study, caution should be used when extrapolating the findings to other populations. Although there was no upper limit of age in this study, the recruited cohort was young. Despite no interaction of the effect by age, applying these findings to older patients should be done with caution. Owing to the nature of the intervention, only the researcher reading the CPET was blinded to study allocation. This may have biased our results. Furthermore, this is a Caucasian predominantly male population. The effect of the intervention may differ in other populations. Because of the possibility of functional capacity decrease during the first 3-month period in the control arm first group, we selected the 3-month Vo_2peak_ as the baseline for this group. However, the effect of the intervention was similar when the same baseline was used for both groups. We used a cross-over design, which may be affected by a carryover effect from 1 period to the next. However, the overall effects of the intervention were similar to that observed after 3 months, before arm cross-over.

## Conclusion

In summary, this pilot randomized cross-over trial shows that smart device-based CR may be a promising alternative for patients unable or unwilling to attend in-person CR, particularly among those with decreased functional capacity. Considering the low cost of the intervention, this may become an alternative in low-resource countries without CR programs and in socially deprived. Considering the low sample size and most being relatively young Caucasian male participants, replication in a larger, more diverse population is needed to determine whether similar effects would be observed. Furthermore, the effectiveness of hybrid CR combining in-person and telerehabilitation needs to be evaluated in future studies.

## Potential Competing Interest

Dr Wohlfahrt reports consulting fees from Servier and Promed; payment or honoraria for lectures, presentations, speaker’s bureaus, manuscript writing or educational events from Servier; support for attending meetings and/or travel from Servier and Promed. Dr Melenovský reports research grants from 10.13039/100009857Regeneron and consulting fees from 10.13039/100004326Bayer, 10.13039/501100004191Novo Nordisk, and 10.13039/100004334Merck. Dr Lopez-Jimenez is a coinventor of several AI algorithms (none related to the current work directly or indirectly) and may benefit commercially when those algorithms are commercialized; participates in Know-How agreements between Mayo Clinic and several IT companies (Kento, WizeCare, K-Health, and Select Research); is a member of the scientific advisory board for Kento, a company that offers home-based cardiac rehabilitation and of the scientific advisory board for Novo Nordisk; reports honoraria for presentations for Asofarma, a pharmaceutical company in Mexico and Latin America in CME conferences; and is a coinventor of several AI-ECG algorithms that have been filed for patenting. Given his role as Editor-in-Chief, Dr Lopez-Jimenez, had no involvement in the peer review of this article and has no access to information regarding its peer review. Full responsibility for the editorial process for this article was delegated to an unaffiliated Editor. The other authors declare no competing interests.
