# Supplementary figures and images for: Remote, Smart Device-Based Cardiac Rehabilitation After Myocardial Infarction: A Pilot, Randomized Cross-Over SmartRehab Study
Source: Mayo Clin Proc Digit Health. 2024 Jun 20;2(3):352–60. doi: 10.1016/j.mcpdig.2024.06.001 (PMC11975814; doi:10.1016/j.mcpdig.2024.06.001)

Supplementary Figure 1

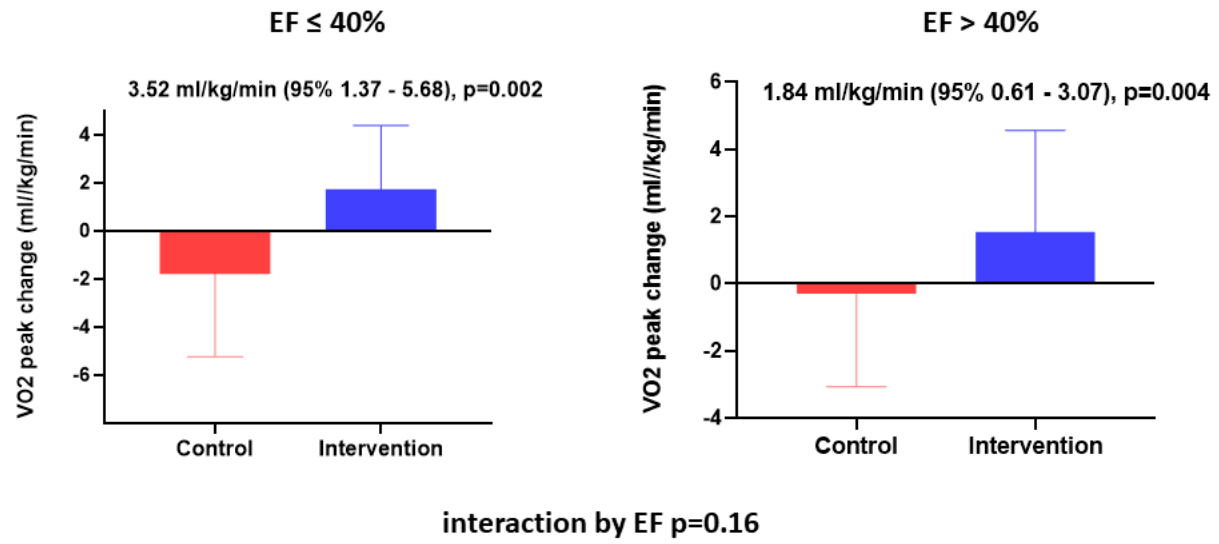

Supplement: Supplemental Figure [file mmc1.pdf]
